# Do household surveys give a coherent view of disability benefit targeting?: a multisurvey latent variable analysis for the older population in Great Britain

**DOI:** 10.1111/rssa.12107

**Published:** 2015-03-03

**Authors:** Ruth Hancock, Marcello Morciano, Stephen Pudney, Francesca Zantomio

**Affiliations:** ^1^ University of East AngliaNorwich; ^2^ University of EssexColchesterUK; ^3^ Ca’ Foscari University of VeniceItaly

**Keywords:** Disability benefits, Disability indices, Multiple surveys

## Abstract

We compare three major UK surveys, the British Household Panel Survey, Family Resources Survey and the English Longitudinal Study of Ageing, in terms of the picture that they give of the relationship between disability and receipt of the *attendance allowance* benefit. Using the different disability indicators that are available in each survey, we use a structural equation approach involving a latent concept of disability in which probabilities of receiving attendance allowance depend on disability. Despite major differences in design, once sample composition has been standardized through statistical matching, the surveys deliver similar results for the model of disability and receipt of attendance allowance. Provided that surveys offer a sufficiently wide range of disability indicators, the detail of disability measurement appears relatively unimportant.

## 1 Introduction

Developed countries like the UK will face severe problems in supporting the projected future growth in the disabled population (McVicar, 2008), and in the older disabled population in particular (Karlsson *et al*., 2006; Organisation for Economic Co‐operation and Development, 2005, Pickard *et al*., 2007). In the UK, there has been a long series of policy reviews by a Royal Commission (Sutherland, 1999), the independent King's Fund (Wanless, 2006), the government (Department of Health, 2009), the Commission on Funding of Care and Support (2011) and various Parliamentary select committees. The current UK Government has recently announced changes to some aspects of the long‐term care funding system (Department of Health, 2013) but debate continues on how best to provide public support to older people with care needs. Such debate and associated policy reform should ideally be evidence based. This requires a robust and accurate baseline picture of the distribution of support for people with disabilities, allowing the development of statistical models to project changes in this picture as levels of disability rise and alternative policy structures are implemented. In turn, this requires good survey data on patterns of disability and receipt of support.

The importance of disability as a policy issue is matched only by the vast range of survey questions that have been used to measure it, and the proliferation of disability indicators across surveys presents difficulties for empirical research. There are many available question designs, supported by limited testing of external validity, internal consistency and test–retest reliability, and some cognitive evaluation of specific question designs (see Sturgis *et al*. (2001) and Jagger *et al*. (2009) for reviews of UK surveys). It is widely recognized that any particular set of disability indicators may give an imperfect description of the concept of disability that is relevant to the analysis and that bias may result from neglect of the measurement error problem (Bound, 1991). However, there has been little cross‐survey comparative work which considers the consistency of the empirical ‘story’ that policy makers would obtain from surveys offering different sets of disability indicators. In practice, researchers often use disability indicators that happen to be available in a survey chosen for convenience or to meet other requirements, and the robustness issue is rarely considered systematically. The green paper (Department of Health, 2009) ‘State of the Nation’ report (Cabinet Office, 2010) and the report of the Commission on Funding Care and Support (2011) are examples of policy documents based on research using a mixture of different survey sources for different purposes.

For policy purposes, we are interested not only in the measurement of disability, but also in its relationship with other key variables like receipt of public support. In this study, we focus on a particular form of public support: the disability‐linked cash benefits which are available to older people. The main disability benefit for people aged 65 years or over in the UK is attendance allowance (AA), which is administered by the Department for Work and Pensions and designed to help to meet the extra costs arising from disability. Besides the age restriction, eligibility for AA requires the claimant to be in need of care to perform daily activities. The AA claim form says ‘you may get Attendance Allowance if your disability means that you need help with your personal care or you need someone to supervise you for your own or someone else's safety’. It defines help with personal care as ‘day‐to‐day help with things like washing (or getting in or out of the bath or shower), dressing, eating, going to and using the toilet, or telling people what you need or making yourself understood’, and supervision as needing ‘someone to watch over you to help you avoid substantial danger to yourself or other people’ (Department for Work and Pensions, 2013). The benefit is not means tested and (in 2012–2013) was worth either £51.85 per week, if care is needed during either the day or night, or £77.45, if care is needed during both. Eligibility for AA is difficult to assess from survey data. In practice, decisions on claims are made by programme administrators on the basis of claimants’ reported health problems and consequent care needs. Once the claim has been made, written evidence is examined by administrative assessors, who can require a medical examination of the claimant. An element of judgement is inevitable, so eligibility is uncertain, even with access to the same information as the administrative assessor. A further challenge is that the information on which the award decision is made is not observable directly in survey data. Rather, surveys offer a set of disability‐related eligibility indicators, from which inference on the success of disability targeting must be drawn. AA is assessed solely on an individual's need for care. It is not means tested (nor taxable) and is unaffected by the presence or circumstances of other household members. So it is possible for more than one household member to receive AA.

Our policy motivation has implications for the appropriate conceptualization of disability. We are not concerned here with medical concepts of impairment, but rather disability conceived as a set of constraints on functioning which originate from health impairments broadly defined. This corresponds to Sen's (1985) ‘capabilities’ approach, which sees the individual choosing a consumption vector *x* from a choice set *X* and a pattern of commodity utilization *f*(·) from a set of possible utilization functions *F*. The individual's chosen vector of ‘functionings’ is *b* = *f*(*x*), which is thus constrained by his or her economic entitlements *X* and available ways of using economic resources *F*. We view the concept of disability as a health‐related limitation on the set *F* relative to some socially agreed minimal norm *N*. The aim of disability policy is to offer support to people for whom *F* ⊂ *N*. Support may take the form of cash or services, both of which expand the individual's choice set *X*, and it may be universal, in which case support is independent of the preintervention *X*, or means tested, in which case entitlement depends on *X*. The important point here is that the concept of disability is concerned with constraints on basic functionings, rather than medical conditions themselves. The survey indicators that are used to measure disability should therefore focus on potential difficulties with everyday activities rather than health or disease.

The contribution of this paper is to investigate whether different indicators of disability, collected in three widely used household surveys, are consistent with a common set of findings relating to the targeting of disability benefits for older people. If we admit the possibility that underlying disability is multi‐dimensional, there are two aspects to this comparability issue: completeness and compatibility. A survey is *complete* in its coverage of disability if its questionnaire content generates disability indicators that are capable of reflecting all the multiple dimensions of disability. Two surveys are mutually *compatible* if their respective indicators of any particular dimension of disability give the same undistorted picture of that underlying concept. For researchers using similar methods but different sources of data to be sure of agreeing on their conclusions, both completeness and compatibility are necessary in general. We investigate three British surveys, the Family Resources Survey (FRS), the English Longitudinal Survey of Ageing (ELSA) and the British Household Panel Survey (BHPS), which have been widely used for research on health, disability and related topics. We find that compatibility is not a serious difficulty, although there are some signs that completeness is a problem for the BHPS.

Typically, the statistical analysis of disability benefit receipt employs a single‐equation framework, in which a variety of disability indicators (or a count index of them) are used as explanatory covariates, together with several other characteristics that are related to socio‐economic status (SES) (see Berthoud and Hancock (2008), Forder and Fernandez (2009) and Zantomio (2013) for examples). Instead, we use a structural equation approach involving a latent concept of disability to study the relationships between disability status, SES characteristics and receipt of AA in the BHPS, ELSA and FRS, at (almost) a single time point, 2002–2003. We assume that an individual's disability status is not directly observable but is reflected in varying degrees by members of a set of imperfect but observable survey indicators. In this respect we follow various researchers since Lee (1982), Van de Ven and Van der Gaag (1982) and Wolfe and Behrman (1984) in considering health status as a latent concept. We assume that the underlying latent disability measure *η* is influenced by a set of SES characteristics and the probability of receiving AA is a function of *η* and SES characteristics. See Bollen (1989) for a review of this class of latent variable simultaneous equation models.

This methodological approach has two major advantages. First, overcoming the arbitrariness of approaches based on a limited set of disability indicators, or a scalar (usually unweighted) count of them, the latent variable framework allows us to develop an index of disability which makes use of all available sample information. This composite index can then be used as a sounder basis for policy analysis focused on the targeting of disability benefit. Second, the latent variable framework reduces the scope for bias arising from the measurement error in observed disability‐related indicators and therefore gives more reliable estimates of the relationship between benefit receipt and influences like disability and income—again improving the robustness of an analysis of benefit targeting. To our knowledge the latent disability approach has not been applied in multiple surveys each with different indicators of disability and the application to disability benefit receipt is also novel.

In Sections 2 and 3 of the paper, we describe the methodological framework and the three surveys, documenting the distributional characteristics of the variables that are used. Results from the model fitted to the full (unmatched) samples are discussed in Section 4. Statistical models are best seen as local approximations, so a comparison of evidence from different surveys may be influenced by differences in sample composition as well as the design of survey instruments. In Section 5 we discuss ways of harmonizing the samples and opt for matching techniques to obtain samples with a (nearly) common distribution for the SES covariates. This reduces the scope of the comparison slightly (the common support constraint) but has the advantage of removing differences due to model approximation errors at the periphery of the region that is covered by the survey samples. In Section 6, we establish the robustness of our findings by examining their sensitivity to various aspects of the analytical approach.

The programs that were used to analyse the data can be obtained from

http://wileyonlinelibrary.com/journal/rss-datasets

## 2 A latent structural equation model of disability status and benefit receipt

In the gerontology literature, Johnson and Wolinsky (1993) conceptualized the dynamics of health status in the older population, viewing functional limitations as outcomes of latent disability. Consistent with this view, we model ‘true’ disability status as an unobservable, possibly multi‐dimensional, phenomenon, which is influenced by socio‐economic characteristics and circumstances. We observe a set of survey indicators, each of which provides a ‘noisy’ measure of underlying disability, satisfying the classical measurement error assumption that all correlation with other socio‐economic characteristics is explained by latent disability. The main outcome of interest, receipt of AA, depends on latent disability and the set of socio‐economic characteristics which influence an individual's propensity to claim and be awarded AA.

Analysis is based on independent samples of $n^{s}$ individuals in surveys *s* = 1, 2, 3. Each sampled individual *i* is characterized by unobserved *Q*‐dimensional ‘true’ disability $\eta_{i}=\left(\eta_{i1}\ldots \eta_{iQ}\right)$, socio‐economic individual characteristics $Z_{i}$ observable in all surveys, a set of survey‐specific disability‐related discrete indicators $D_{ij}^{S},j=1,\ldots ,J^{S}$, and a binary indicator of benefit receipt ($R_{i}=1$) or non‐receipt ($R_{i}=0$). We aim to draw inferences about the conditional distributions *P*(***η***|**Z**) and *P*(*R*|***η***,**Z**) which describe respectively the distribution of disability in the population and the relationship between benefit receipt and the individual's disability and other characteristics. By definition, these population distributions are independent of any survey that is used to draw inferences about them. An important question is whether the distributions $P^{s}\left(R,D_{1}^{s}\ldots D_{J^{s}}^{s}|Z\right)$ that are produced by the three surveys with their different disability indicators nevertheless give a coherent indication of underlying ‘true’ disability ***η*** and its relationship with benefit receipt *R*.

We estimate a structural equation model which comprises three components: a survey‐specific measurement model, a disability model and a benefit receipt model. We use an ordinal quasi‐linear structure for disability measurement:

(1) $${\tilde{D}}_{ij}^{s}=\alpha_{j}^{s}+\lambda_{j1}^{s}\eta_{i1}+\;\;\ldots \;+\lambda_{jQ}^{s}\eta_{iQ}+\varepsilon_{ij}^{s},$$

(2) $$D_{ij}^{s}=m\;\text{if and only if}\;A_{jm-1}^{s}⩽{\tilde{D}}_{ij}^{s}<A_{jm}^{s},.9pcm=1,\ldots ,M_{j}^{s},$$

where the coefficients $\lambda_{jq}^{s}$ are factor loadings relating observed indicators in survey *s* to underlying disability, $\varepsilon_{ij}^{s}$ is a normally distributed residual term representing random‐response error, implying an ordered probit link function generating the observable indicator $D_{ij}^{s}$ from its unobserved continuous form ${\tilde{D}}_{ij}^{s}$, $M_{j}^{s}$ is the number of response categories for indicator $D_{ij}^{s}$ and the $A_{jm}^{s}$ are threshold parameters. In what follows we refer to equations (1) and (2) as the measurement model. The *q*th disability component $\eta_{iq}$ is related to $Z_{i}$ through a linear relationship representing the processes leading to disability (disability model):

(3) $$\eta_{iq}=\theta_{q}Z_{i}+\nu_{iq}$$

where $\theta_{q}$ is a vector of coefficients. The residual $\nu_{iq}$ captures other unobservable factors and satisfies. $E(\nu_{iq}|Z_{i})=0$. Benefit receipt is modelled by a probit specification (the benefit receipt model):

(4) $${\tilde{R}}_{i}=\beta Z_{i}+\gamma_{1}\eta_{i1}+\;\;\ldots \;+\gamma_{Q}\eta_{iQ}+u_{i}$$

where the observed benefit receipt status $R_{i}=1$ when ${\tilde{R}}_{i}>0$ and $R_{i}=0$ otherwise, ***β*** and the $\gamma_{q}$ are coefficients and $u_{i}$ is a stochastic disturbance term. Although allowing correlation between the *Q* latent constructs, we make the standard assumption underlying probit models that the stochastic residual $u_{i}$ is independent of ($Z_{i},\eta_{i}$) and the residuals in the measurement equations (1). In writing equations (3) and (4), we allow the same covariates to represent the influences on disability and on benefit claim behaviour. This is not necessary, and there may be exclusion restrictions (which are not necessary for identification) on the vectors ***β*** and $\theta_{q}$.

We say that survey *s* is *complete* if the *J*×*Q* loadings matrix $\left\{\lambda_{jq}^{s}\right\}$ is of full column rank *Q*; this requires that, for each dimension of disability *q*, at least one of the *j* observed indicators $D_{ij}^{s}$ has a non‐zero loading $\lambda_{jq}^{s}$. In the on‐line appendix, we show that completeness is sufficient to identify the model under our assumptions. The surveys are said to be *compatible* if the assumption of common parameters across surveys in equations (3) and (4) is valid.

Several studies have shown that, in the older population, women tend to report significantly higher rates of functional difficulties than comparable men (Rahman and Liu, 2000; Crimmins *et al*., 2011). Some researchers have attributed this apparent female functional disadvantage to higher true prevalence of non‐fatal but disabling conditions such as arthritis and osteoporosis (Wingard, 1984; Verbrugge and Wingard, 1987). Others have found that, even when controlling for chronic conditions, women still report higher mean levels of functional disability. This could be due to a higher propensity for women to report ill health than men with the same underlying true health status (Verbrugge, 1980; Hibbard and Pope, 1983), or to heightened sensitivity to symptoms because of gender‐specific social expectations and life experience (Verbrugge and Wingard, 1987) or to task specificity if women are more engaged than men in household tasks that require actions such as bending and lifting. This measurement issue has been termed variously ‘state‐dependent reporting bias’ (Kerkhofs and Lindeboom, 1995), ‘scale of reference bias’ (Groot, 2000) and ‘response category cut‐point shift’ (Lindeboom and van Doorslaer, 2004). However, unless we can specify *a priori* a subset of indicators in each survey for which response behaviour is gender invariant, it is impossible to distinguish the causal effect of gender on true latent disability from its effect on reporting behaviour. We allow for the possibility of inherent gender differences in disabilities by allowing the parameters of the measurement equations (1), (2) to be gender specific. We therefore exclude gender from $Z_{i}$ of equation (3).

We estimate the system comprising all equations (1), (2), (3), (4) simultaneously allowing for the discrete nature of the dependent variables, using robust maximum likelihood as implemented in *MPlus* version 6.11 (Muthén and Muthén, 2010). This is done separately for each survey, to avoid imposing by assumption any homogeneity across surveys. All standard errors are clustered by household to allow for intrahousehold correlation. However, since we do not have access to indicators of the geographical primary sampling units that were used in the sampling designs in the FRS, we cannot allow for geographical clustering, and the standard errors quoted are expected to understate sampling variation to a small extent. We have been able to confirm this for the ELSA sample, where primary sampling unit and stratum indicators are available; standard errors increase to a negligible extent (details are available on request). This suggests that the true size of our tests of between‐survey parameter stability is very slightly larger than the nominal level of significance, giving a small tendency to overreject parameter stability, which increases the force of our eventual conclusions.

## 3 Data

The analysis is based on three sample surveys: the first wave of the ELSA, the corresponding 12th wave of the BHPS and the 2002–2003 cross‐section of the FRS. All three surveys have been widely used for research on physical health and disability: see, for example, Melzer *et al*. (2005), Banks *et al*. (2006), Mayhew *et al*. (2010) and Chan *et al*. (2012) for the ELSA, Benítez‐Silva *et al*. (2009), Oswald and Powdthavee (2008) and Banks *et al*. (2009) for the BHPS, and Kasparova *et al*. (2007), Hancock and Pudney (2013) and Morciano *et al*. (2014) for the FRS. Although the three surveys are broadly similar in sampling design, they differ considerably in their initial response and degree of cumulated attrition, and in methods of constructing weights that are intended to deal with departures from uniform sampling; Table 1 gives a summary of these differences.

**Table 1:** Comparing the FRS, ELSA and BHPS along sample design and structure, data collection and weighting procedures†

	*FRS (2002–2003)*	*ELSA (wave 1)*	*BHPS (wave 12)*
Population coverage	People in private dwellings, UK	People in private dwellings, England	People in private dwellings, Great Britain
Timing	Cross‐section, April 2002–March 2003	Longitudinal study, March 2002–March 2003	Longitudinal study, September 2002–December 2002
Frame	Royal Mail's small users’ *Postcode Address File*	1998, 1999 and 2001 HSE, samples drawn from different vintages of the *Postcode Address File*; the ELSA includes households with an adult of 50 years or older who agreed to re‐contact	*Postcode Address File*
Sample design	Sample design is an equal probability selection mechanism, with two‐stage stratified random sampling	Two‐stage stratified equal probability selection mechanism design in the HSE	Two‐stage stratified equal probability selection mechanism design at wave 1 (1991)
Stratification variables	Region, socio‐economic group profile, adult economic activity rate, male unemployment rate	Health authority, proportion of households with a head of household in a non‐manual occupation	Region, socio‐economic group profile, proportion of pensionable age, proportion of employed people working in agriculture
Response rate	64%	HSE response rate 69%; 92% consent to be contacted for the ELSA; 70% response rate at ELSA wave 1, giving 44% response overall	74% at wave 1; 50% allowing for cumulated attrition to wave 12
Weighting	*Design weights* adjust for selection of households within addresses; *non‐response weighting* is not used; *calibration weights* are based on age, gender, lone parents or all families with children, housing tenure and council tax band distributions from official statistics	*Non‐response weights* compensate for unit non‐response at HSE, refusal post HSE and non‐response in ELSA wave 1; ELSA phase uses inverse response probability from a logistic regression on age of the oldest household member, regional health authority, household size, social class, year of HSE interview and long‐standing illness, as observed in HSE data sets; *calibration weights* match age–sex cell frequencies from the non‐institutionalized population of the 2001 census	*Design weights* adjust for selection of households within addresses; *non‐response weights* at household level based on region, socio‐economic group and type of accommodation; at individual level, inverse response probability from logistic regression on region, housing tenure, affluence, household size, marital and employment status, age, sex; *calibration weights* use 1991 and 2001 census marginal distributions for household tenure, household size, number of cars, age and sex
Question wording on AA receipt	‘And looking at this card, are you at present receiving any of the state benefits shown on this card—either in your own right or on behalf of someone else in your household?’	‘Have you/you or your husband/wife/ partner received any of these health or disability benefits in the last year?’ ‘Which of these health or disability benefits have you received in the last year?’ ‘Which of these health or disability benefits are you receiving at the moment?’	‘I am going to show you four cards listing different types of income and payments. Please look at this card and tell me if, since September 1st 2001, you have received any of the types of income or payments shown, either just yourself or jointly?’

†Source: Campbell (2004), Taylor *et al*. (2003, 2006, 2007) and Lound and Broad (2013).

The FRS has a sample size of over 25000 private households. It is an annual cross‐section and therefore suffers from non‐response but not accumulated attrition. The FRS response rate in 2002–2003 was 64% of eligible households (Campbell, 2004). The BHPS started in 1991 and followed a sample of approximately 10000 households annually, so our sample has come through 12 waves of attrition and possible panel conditioning. The initial BHPS response rate was 74% and 67% of those original respondents gave a full interview in wave 12 (Lynn *et al*., 2006). The ELSA is a panel of individuals aged 50 years and older and their partners in approximately 8000 private households in England. Panel membership is based on interview in the 1998, 1999 or 2001 Health Surveys for England (HSEs). The wave 1 ELSA data are thus potentially affected by non‐response in the HSE and a further round of attrition; HSE response rates were 74% (1998), 76% (1999) and 74% (2001) and, of those selected for the ELSA, around 70% responded to its first wave (Taylor *et al*., 2003). We choose the first wave of the ELSA as our common time point to avoid the effects of subsequent attrition. We limit our analysis to people aged 65 years or over, living in England. The former restriction is because only people aged 65 years or over can claim AA. The latter is imposed by the ELSA sampling frame. We also exclude respondents receiving disability living allowance (which is a similar benefit that can be claimed before age 65 years) because disability living allowance recipients cannot also claim AA.

The three surveys also differ in questionnaire content. The FRS collects very detailed income and benefit information, used as the basis for most official statistics on welfare and disability programme targeting, but a limited set of disability indicators. The ELSA provides a richer range of health and disability measures but slightly more limited income data than the FRS (for example, the ELSA collects some income components gross of tax and others net). In the BHPS, it is not always possible to distinguish whether a particular source of income is gross or net. BHPS information on health and disability is more detailed than the FRS in some respects but less so than the ELSA. The surveys differ in the information that they collect by proxy for participants who cannot provide responses themselves; in particular the FRS collects information on disability and AA receipt from proxy respondents, whereas the BHPS and ELSA do not. We return to treatment of proxy respondents below. Campbell (2004), Taylor *et al*. (2003) and Taylor *et al*. (2006) have respectively given detailed descriptions of FRS, ELSA and BHPS sample design and data collection procedures.

In each survey, information about receipt of AA, recorded by the binary variable *R* ${}_{i}$, is collected through questions following those on health and disability. Thus, none of the three surveys is especially vulnerable to the justification bias in disability measurement that is a concern when the benefits module precedes the health module within the questionnaire (Crossley and Kennedy, 2002). There are differences in the reference period for questions on AA receipt: the BHPS covers the year preceding the interview; the FRS refers specifically to the time of interview; and the ELSA asks separately about different reference points. For the ELSA we use receipt of AA at the time of interview, to give comparability with the FRS.

A wide range of disability indicators is available in one or more of the three surveys. In this study, we use subjective indicators which are the most widely available in social surveys. On‐line appendix Table O1 reports the functional limitation indicators $D_{j}$ that are offered by each survey and used in our analysis, with their prevalence rates among AA recipients and non‐recipients. Binary indicators in the FRS cover difficulties in eight areas of life. The ELSA provides a longer list of indicators including limitations to specific activities of daily living (ADLs) (Katz *et al*., 1963) or instrumental activities of daily living (IADLs) (Lawton and Brody, 1969). The BHPS indicators include binary variables representing activities that are limited by health and a set of six‐point categorical variables, built from two questions on whether the respondent usually manages to perform a set of mobility and personal care activities alone or only with assistance, and whether he or she finds it very easy, fairly easy, fairly difficult or very difficult. There is a considerably higher sample prevalence of reported functional limitations among AA recipients than non‐recipients, consistently across surveys and specific indicators.

The choice of other personal characteristics included in **Z** is governed by previous work on the socio‐economic gradient in health or disability (e.g. Goldman (2001)) and on older people's benefit claim behaviour (e.g. Zantomio (2013) in relation to AA, and Hernandez *et al*. (2007) and Pudney *et al*. (2006) for means‐tested benefits). We use age (in the form of a spline with a knot at the median age across all samples of 73 years to allow for non‐linearity in the age gradient of disability and receipt of AA), gender, being educated beyond the compulsory minimum, housing tenure and log‐equivalized pre‐benefit income in both equations. Information on past occupation is not collected from pensioners in the FRS and therefore is not included in **Z**. Income represents both the socio‐economic gradient in health and the basic need for financial support which underlies benefit claim behaviour. It is derived as the sum of income from pensions, earnings, savings and other sources received by any member of the benefit unit (defined as an adult plus their spouse (if applicable) plus any dependent children whom they are living with), but it excludes disability and means‐tested benefits. Disability benefits must be excluded from the latent disability equation because they are a consequence, and not a cause, of disability, and from the AA equation as it is income in the absence of AA that influences the decision to claim. Means‐tested benefits are excluded because their level can also depend on disability through the severe disability premium, which is an addition to the income thresholds that are used to assess entitlement to means‐tested welfare benefits and applies where the claimant is receiving AA. To account for differences in benefit unit size we apply the modified Organisation for Economic Co‐operation and Development equivalence scale to income. For this older population, our income measure is dominated by pension income, which is a good indicator of past labour market success, itself strongly related to lifestyle characteristics which have associated health implications. Thus estimates of the effect of income on disability should be interpreted in this wide sense. Log‐income is entered as a spline with a knot at the median log‐income level ( log (£615.70) per month, 2002 prices). Our definition of housing tenure distinguishes those who own their homes outright from those who rent or are still repaying their mortgage. Outright home ownership is used to capture an additional long‐term socio‐economic influence on health. It also allows for the lower financial need (lower housing costs) that outright owners have compared with those who face rent or mortgage costs, to influence their benefit claim behaviour. Current partnership status (married or cohabiting *versus* single) is also included as a covariate in the AA receipt equation since it has previously been found to affect benefit claim behaviour (Hernandez *et al*., 2007; Pudney *et al*., 2006).

All variables have been derived in a consistent manner as far as possible, although perfect comparability cannot be guaranteed (sample means and standard deviations for the socio‐economic characteristics **Z** that are observed in each sample are given in Table O3 of the on‐line appendix). There are some differences between surveys (for the subsamples aged 65 years and older): for example, before survey weights are applied the ELSA sample members are slightly younger and more educated than their BHPS and FRS counterparts, the proportion of outright homeowners is higher in the ELSA and the BHPS than in the FRS, and the mean of (log‐) income is significantly higher in the BHPS than in the ELSA and the FRS. The FRS reports a higher rate of AA receipt (9.7%) than does the ELSA or BHPS (7.2%). Comparisons with administrative data are not straightforward because they include AA recipients in the care home population. We estimate that, of the over‐65‐years non‐care‐home population, excluding those who received disability living allowance, between 12.7% and 13.8% received AA in 2002. This is based on Department for Work and Pensions statistics on recipients of AA and disability living allowance which include, but do not separately distinguish, recipients in care homes, together with estimates from Comas‐Herrera *et al*. (2010) on the numbers of over 65‐year‐old residents in care homes and the proportions of them who receive public support with the care home fees and are therefore not eligible to receive AA. All three surveys therefore seem to underrepresent AA recipients but the FRS less so than the ELSA or BHPS.

Ideally we would use all proxy cases since they are likely to include some of the most severely disabled respondents. This view is supported by an analysis of proxy respondents in the FRS, revealing AA receipt to be about twice as high among proxy respondents as non‐proxy respondents (18.1% against 9.1%). However, we are forced to exclude proxy responses in the ELSA (1.9%) and BHPS (4.1%) as their proxy questionnaires do not collect the respondent's disability (ELSA) or AA receipt (BHPS). We retain the larger proportion of proxy cases (6.5%) in the FRS which does collect this and other relevant information for proxy cases, using a proxy response as an additional disability indicator in the measurement model. After these exclusions and dropping cases with missing values for variables that are used in the analysis, the sample sizes are 1042, 5142 and 6744 individuals from the BHPS, ELSA and FRS respectively. We also assess the sensitivity of the results to the exclusion of FRS proxy cases in which case the FRS sample is reduced to 6308.

In the next section, we present results based on the full unweighted samples, and we return to the issue of sample comparability in Section 5.

## 4 Estimation results

### 4.1 The measurement model

To implement the model, we must specify the dimensionality of latent disability and choose a normalization to deal with its non‐observability and lack of natural units of measurement. Our main results come from survey‐specific structural equation models with a single latent disability factor and a simple normalization. For the latter, we choose *a priori* one indicator from each survey that appears to be based on essentially the same question. These are the FRS question about mobility (‘moving about’), the ELSA question about capacity to ‘walk 100 yards’ and the BHPS question about ‘walking more than 10 minutes’. We then normalize the factor loading for each of these indicators to be 1. In Section 6, we explore the sensitivity of the results to our choice of normalization and number of factors. Controversy exists over whether functional disability should be treated as a one‐dimensional or multi‐dimensional construct (see for instance Fitzgerald *et al*. (1993) and Spector and Fleishman (1998). As a check on the robustness of our main model, in Section 6 we also estimate a two‐factor model distinguishing physical and cognitive disabilities. Although passing reference is often made to the multi‐dimensional nature of disability, we are not aware of any previous estimates of multifactor models of this kind in the existing literature.

The estimates of the measurement model are presented in the on‐line appendix Table A1: the factor loadings $\lambda_{jq}^{s}$, representing the effect of latent disability *η* on each indicator $D_{ij}^{s}$, are positive and highly significant in each survey. Although the pattern of estimated factor loadings is similar for male and female respondents in each survey, there are significant differences. In the FRS, the loading that is associated with ‘lifting, carrying or moving objects’ is significantly higher for women. In the ELSA, factor loadings associated with reported difficulties in ADLs like ‘bathing or showering’, ‘eating’, ‘getting in or out of bed’ and ‘using the toilet’ and IADLs like ‘doing work around the house or garden’ are significantly lower for women; in the BHPS, a significantly lower factor loading for women is also found for difficulties in bed transfers and ‘bathing or showering’. The Akaike information criterion suggests that the unrestricted models (which allow the parameters of the measurement equations (3) to be gender specific) provide slightly better balances of model fit and parsimony. This result is also confirmed by the Satorra‐Bentler (2001) test at the 1% level for each of the three surveys.

### 4.2 The disability model

Estimates for the model (3) of latent disability status are reported in Table 2, together with *t*‐tests of individual coefficient equality and the overall $\chi^{2}$ Wald tests for equality of the whole coefficient vector for each pair of surveys. The conditional mean of latent disability *η* increases with age: the FRS and ELSA display a non‐linear relationship between age and disability, with a higher gradient beyond age 73 years. In the BHPS we find a strong and near‐linear relationship between age and disability. Higher education and pre‐benefit income are associated with lower disability, giving evidence of a socio‐economic gradient in disability that is consistent across surveys. Being a homeowner decreases the conditional mean of *η*, particularly in the ELSA. The variance of the latent disability factor is greater in the BHPS than in the FRS or ELSA, but we find that the factor variances are quite comparable across surveys (a 10% significant difference is found only for the FRS–ELSA contrast). The estimated coefficients for the FRS and ELSA are similar in size and the Wald test cannot reject the hypothesis of equality; when the BHPS is used as the basis for comparison, the null hypothesis of joint equality of coefficients is rejected (*p*‐values 0.064 and 0.028).

**Table 2:** Estimates of the latent disability equation†

*Covariate*	*Coefficients*			*Tests and coefficient differences*		
	*FRS*	*ELSA*	*BHPS*	*FRS–ELSA*	*FRS–BHPS*	*ELSA–BHPS*
Spline age 65–73 years	0.038‡	0.035‡	0.127‡	0.003	−0.089‡	−0.092‡
	(0.013)	(0.012)	(0.036)	(0.018)	(0.038)	(0.038)
Spline from age 73 years onwards	0.091‡	0.099‡	0.128‡	−0.008	−0.037§	−0.029
	(0.008)	(0.008)	(0.020)	(0.011)	(0.022)	(0.022)
Post‐compulsory education	−0.279‡	−0.28‡	−0.182	0.001	−0.096	−0.097
	(0.065)	(0.061)	(0.149)	(0.089)	(0.163)	(0.161)
Income spline to median	−0.162‡	−0.046	−0.172§	−0.116§	0.009	0.125
	(0.047)	(0.052)	(0.104)	(0.070)	(0.114)	(0.116)
Income spline from median	−0.336‡	−0.310‡	−0.558‡	−0.025	0.223	0.248
	(0.085)	(0.072)	(0.206)	(0.111)	(0.223)	(0.218)
Outright owner	−0.382‡	−0.487‡	−0.185	0.105	−0.197	−0.302§
	(0.064)	(0.064)	(0.151)	(0.090)	(0.164)	(0.163)
Variance $\left(\sigma_{v}^{2}\right)$	3.012‡	2.543‡	3.298‡	0.469§	−0.286	−0.755
	(0.275)	(0.225)	(0.788)	(1.320)	(0.343)	(0.921)
	*Sample size*			*Coefficient equality* $\chi^{2}$ *(6)*		
	6744	5142	1042	4.361	11.920§	14.139§§

†Standard errors are given in parentheses. ‡Statistical significance of the coefficient, *t*‐test cross‐sample coefficient difference and $\chi^{2}$‐statistic: *p*<0.01. §Statistical significance of the coefficient, *t*‐test cross‐sample coefficient difference and $\chi^{2}$‐statistic: *p*<0.1. §§Statistical significance of the coefficient, *t*‐test cross‐sample coefficient difference and $\chi^{2}$‐statistic: *p*<0.05.

### 4.3 The benefit receipt model

**Figure 1:**
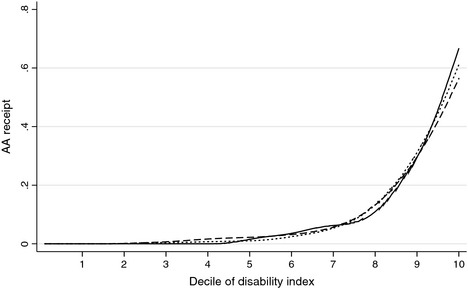
Proportion of people in receipt of AA by predicted severity of disability (the bandwidth was set equal to 0.4): smoothed local linear regressions applied to the FRS (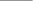), ELSA (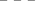) and the BHPS (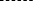) samples

Estimates for equation (4), describing the relationship of AA receipt with socio‐economic characteristics and latent disability, are reported in Table 3. Receipt of AA is clearly disability related in each of the surveys, and disability consistently emerges as the dominant variable in explaining AA receipt. Although disability might raise barriers to claiming and at the same time reduce individuals’ capacity to benefit from additional cash income, the survey evidence suggests that there is successful targeting of AA on the disabled older population, irrespective of the source of survey data. This is clear from Fig. 1, which shows the mean prevalence of AA receipt within each decile of the distribution of the posterior prediction of latent disability for each individual. The strong disability targeting of AA emerges very clearly for all three surveys.

**Table 3:** Estimates of the equation for receipt of AA†

*Covariate*	*Coefficients*			*Coefficient differences*		
	*FRS*	*ELSA*	*BHPS*	*FRS‐ELSA*	*FRS‐BHPS*	*ELSA‐BHPS*
Latent disability *η*	0.569‡	0.477‡	0.538‡	0.092§	0.031	−0.060
	(0.041)	(0.035)	(0.095)	(0.054)	(0.103)	(0.101)
Female	0.122§	0.251‡	−0.068	−0.129	0.190	0.319§
	(0.065)	(0.073)	(0.172)	(0.098)	(0.184)	(0.187)
Spline age 65–73 years	−0.040‡	−0.036‡	−0.084‡	−0.004	0.043§	0.048§
	(0.008)	(0.007)	(0.021)	(0.011)	(0.022)	(0.022)
Spline from age 73 years onwards	0.058‡	0.046‡	0.028§	0.012	0.030§	0.017
	(0.006)	(0.007)	(0.015)	(0.009)	(0.016)	(0.016)
Post‐compulsory education	−0.161§§	−0.238‡	−0.070	0.077	−0.090	−0.167
	(0.065)	(0.071)	(0.155)	(0.096)	(0.168)	(0.171)
(log‐) income spline to median	−0.008	−0.092§	−0.041	0.083	0.033	−0.050
	(0.048)	(0.049)	(0.090)	(0.069)	(0.102)	(0.102)
(log‐) income spline from median	−0.392‡	−0.422‡	−0.411§	0.030	0.019	−0.011
	(0.120)	(0.154)	(0.247)	(0.195)	(0.274)	(0.291)
Outright owner	−0.136§§	−0.006	−0.265	−0.130	0.128	0.259
	(0.062)	(0.071)	(0.164)	(0.095)	(0.175)	(0.178)
Married or cohabiting	−0.076	0.087	−0.171	−0.163	0.094	0.257
	(0.064)	(0.076)	(0.182)	(0.100)	(0.193)	(0.198)
$\chi^{2}$(9) test of coefficient equality				14.398	14.685§	14.844§

†Standard errors are given in parentheses. ‡Statistical significance of the coefficient, *t*‐test cross‐sample coefficient difference and $\chi^{2}$‐statistic: *p*<0.01. §Statistical significance of the coefficient, *t*‐test cross‐sample coefficient difference and $\chi^{2}$‐statistic: *p*<0.1. §§Statistical significance of the coefficient, *t*‐test cross‐sample coefficient difference and $\chi^{2}$‐statistic: *p*<0.05.

The estimated probability of receiving AA declines non‐linearly with income. We find that, below median income, the coefficient is significant at the 10% level only in the ELSA, so the income gradient in AA receipt operates primarily among higher income people. The negative gradient is due both to the low incidence of disability among high income groups (Pudney, 2010) and to the low propensity of these groups to claim benefit (Hernandez *et al*., 2007). Consequently, although AA is not means tested, patterns of receipt mimic to some degree the effect of means testing for those in the top half of the pensioner income distribution.

We find significant evidence of a negative association between the level of education and AA receipt in both the ELSA and the FRS. This suggests that any advantage that more‐educated people may have in navigating the benefits system is outweighed by factors such as less contact throughout their lives with the benefit system, or greater perceived stigma from claiming benefits (as also found in Zantomio (2013)). Owning one's home outright reduces significantly the probability of AA receipt in the FRS and the BHPS. This could reflect a lower financial need among homeowners, or the same factors that may be at work for more‐educated people could play a similar role for outright homeowners.

Receipt of AA appears gender related in the FRS and the ELSA, where men are less likely to receive AA than women; gender differences are insignificant in the BHPS. In all three surveys, age affects the probability of AA receipt non‐linearly, with a convex age profile. There is again a significant difference between the estimated age profile for the BHPS compared with the FRS and ELSA, with a less significant upturn at older ages. Finally, none of the surveys suggests that the presence of a partner significantly affects the probability of receiving AA. Inspection of coefficients in this piecemeal way creates a bias in favour of finding significant differences, because of the multiple comparisons that are involved. However, a joint Wald test finds a significant difference between the BHPS and the other two samples (*p*‐values 0.100 and 0.095). We do not reject coefficient equality between the FRS and ELSA.

**Figure 2:**
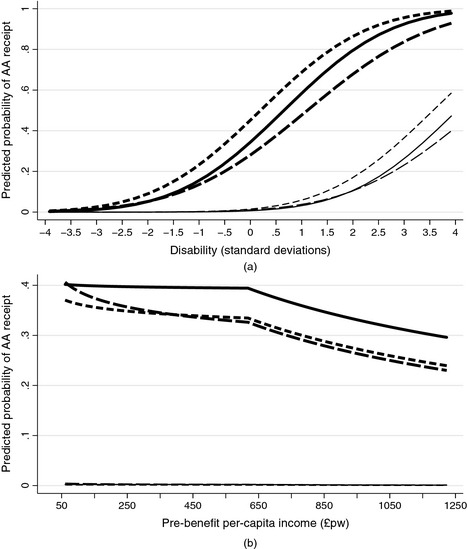
Predicted probabilities of AA receipt by survey for two benchmark cases: (a) AA–disability relationship (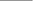, high income younger couple, FRS; 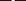, high income younger couple, ELSA; 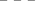, high income younger couple, BHPS; 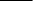, low income older widow, FRS; 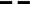, low income older widow, ELSA; 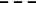, low income older widow, BHPS); (b) AA–income relationship (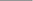, low disability younger couple, FRS; 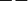, low disability younger couple, ELSA; 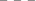, low disability younger couple, BHPS; 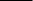 high disability older widow, FRS; 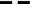, high disability older widow, ELSA; 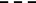, high disability older widow, BHPS)

In Fig. 2(a), we compare the implications of the estimated models, for two illustrative individuals: a 65‐year‐old man living with his partner as an outright homeowner with income 50% above the median and an 85‐year‐old non‐homeowner widow, with equivalized income 75% of the median. Both have compulsory minimum education. In Fig. 2(a), the between‐survey differences in their AA disability profiles are modest in comparison with the predicted differences between hypothetical individual types. For example, at a level of disability 1 standard deviation above the mean, the three models predict a 4–7% rate of receipt for the couple compared with a 50–71% rate for the widow. At a level of disability of 2.5 standard deviations above the mean, the ranges are 16–26% for the couple and 77–92% for the widow.

In Fig. 2(b), we compare the estimated AA income profiles. Again, the between‐survey differences in these profiles are modest in comparison with the predicted differences between hypothetical individual types. The rate of receipt for the low disability (at the 25th percentile of the disability index distribution) couple is essentially 0, whereas the rate of receipt for the high disability type (at the 75th percentile of the disability index distribution) ranges from 31% to 37% in the income interval that we consider. The rate of receipt is non‐linear in income: almost flat below the median equivalized income and steadily declining thereafter. For example, the rate of receipt for the highly disabled widow ranges from 34% to 39% at the 25th (£435 per month) and at the 50th percentile of the income distribution, and 27–33% at the 75th percentile (£917 per month).

In general, the three surveys show similar patterns in terms of their empirical AA–disability relationship. However, at some levels of disability between‐survey differences in predicted probabilities of AA receipt are sizable. The between‐survey differences are statistically significant when the BHPS is used as the basis for comparison. In the next section we investigate the extent to which these differences might be attributable to differences in sample composition.

## 5 Controlling sample composition

If statistical models are empirical approximations local to the region spanned by the sample data, then cross‐survey differences in model estimates might result only from differences in their covariate distributions rather than any more fundamental measurement problem. As Table O2 in the on‐line appendix makes clear, there are important differences in the empirical distribution of the covariates in the three surveys, resulting from the differences in design and patterns of response.

In single‐survey analysis, the standard method of controlling sample composition is to use survey weights. Broadly, these have three elements: *design weights* which compensate for deliberate non‐uniform sampling rates across the population, *non‐response weights* which compensate for variations in response probabilities across individuals and households with different characteristics, and *calibration* or *post‐stratification* weights used as a final step to bring the sample composition in line with whatever is known about the structure of the population. If the assumptions underlying the derivation of weights (e.g. missingness at random) are valid and if the weights are implemented in the ‘correct’ way by each survey, then separate weighted samples should identify essentially the same population parameters, if the questionnaires have the same informational content. However, the weighting strategies are not harmonized across the three surveys (see Table 1 for a summary of the weighting procedures). Different covariates appear in response models that are used to generate non‐response weights and the calibration stage is done in different ways. Given these methodological conflicts, it is unlikely that the use of the weights supplied with each survey will solve the comparability problem, and it is even possible for weighting to impair, rather than to improve, comparability. Nevertheless, we have carried out weighted analyses and found that weighting does not fully eliminate the between‐survey differences that we found in Section 4. For the disability equations, the Wald $\chi^{2}$ of coefficient equality *p*‐value slightly rises from 0.064 to 0.102 for the FRS–BHPS comparison and it decreases from 0.028 to 0.026 for the ELSA–BHPS comparison (see on‐line appendix Table A2). For the AA equation, the Wald $\chi^{2}$ *p*‐value decreases from 0.100 to 0.048 for the FRS–BHPS comparison and it rises from 0.095 to 0.142 for the ELSA–BHPS comparison (see on‐line appendix Table A3).

Matching techniques provide another way of reducing bias from differences in the sampling distribution of covariates across surveys. They involve estimating the models by using survey‐specific subsamples which are balanced in terms of the set of common covariates that are thought to influence disability and AA receipt. The matching approach has not been widely used in this context, but there are some precedents (Rosenbaum, 2002; D'Orazio *et al*., 2006; Rässler, 2002). The method requires (at least partial) common support across surveys for the matching variables, which holds in our samples (see Table O2 of the on‐line appendix). We make the assumption that the matching variables are comprehensive in the sense that, conditionally on them, subsample selection can be regarded as random. This is essentially the same missingness at random assumption underlying weighting methods and, although untestable, is plausible, given the three surveys’ sample design.

In practice, we take each survey in turn as a baseline and construct matched subsamples from the other two surveys, yielding six pairs of matched samples. The matching algorithm (Leuven and Sianesi, 2003) uses one‐to‐one nearest neighbour matching, minimizing the Mahalanobis distance for the variables age, gender, post‐compulsory education, partnership, housing tenure and log‐pre‐benefit net income. Matching is performed without replacement, to avoid repeated use of the same observation from the matched survey, at the cost of possibly reducing the size of successfully matched samples. According to the available sample size, in each round of pairwise matching we impose a caliper (ranging from 0.04 to 0.5) to prevent poor matches, equivalent in practice to exact matching of binary variables and very close matching for the continuous income and age variables; *t*‐tests for the equality of means between each baseline sample and the corresponding matched samples were used to confirm the success of the algorithm in balancing the conditioning covariates. We also discarded matched pairs of observations whose income difference was in the top 5% when matching the BHPS to the ELSA and the top 10% when matching the ELSA to the BHPS. Means of socio‐economic variables and AA receipt in the matched samples are given in Table O3 of the on‐line appendix.

We repeated estimation of the system of equations (1), (3) and (4) on each of the six pairs of matched samples. Results obtained for the measurement equations (1), (2) confirm the patterns that were described in Section 4, with mobility indicators playing a dominant role as indicators of latent disability. The three panels of on‐line appendix Table A4 report estimated regression coefficients for the latent disability equation (3) obtained from samples mimicking the FRS, ELSA and BHPS sample compositions respectively. As in the unmatched samples (Table 2), we obtain significant disability gradients in age (positive) and income (negative) consistently across surveys, although some coefficients lose significance in smaller samples. Using separate *t*‐tests of cross‐sample coefficient stability, we would reject the null hypothesis of coefficient equality only for the first spline of income coefficient (at the nominal 5% level), when the FRS or ELSA are used to mimic the BHPS sample composition. However, none of the individual *t*‐tests would be significant if a Bonferroni correction were used, and the striking similarity of estimated coefficients is confirmed by the $\chi^{2}$‐tests of coefficients’ joint equality: in none of the six paired survey comparisons is the null hypothesis rejected.

Estimated coefficients for the AA receipt equation (4) are reported in on‐line appendix Table A5. The positive disability gradient in AA receipt found in the unmatched samples (Table 3) is also evident in the matched samples: estimates for the disability coefficient *γ* are positive, significant and remarkably similar in size. The negative income gradient is also confirmed, except for an insignificant positive coefficient when the ELSA mimics the BHPS sample composition. The negative association between homeownership and receipt of AA is again found whenever the coefficient on homeownership is significant. For age, coefficient equality is rejected at the 5% level only for the second spline when BHPS observations are used to mimic the ELSA sample composition; but such isolated rejections are likely to arise from sampling error when large numbers of individual *t*‐tests are used, and none would be significant if a Bonferroni correction were used. Joint Wald $\chi^{2}$‐tests of coefficient equality again fail to reject the hypothesis of coefficient equality in any of the six pairwise comparisons.

## 6 Robustness

### 6.1 The number of factors

In the estimated one‐factor measurement models of on‐line appendix Table A1, there is a strikingly low correlation between the latent disability index and those indicators which might be thought to represent cognitive rather than physical disability. To allow for a distinction between physical and cognitive disability, we have also estimated a two‐factor model for each sample, following an exploratory factor analysis of the disability indicators. The attempt failed for the BHPS, where only a single factor could be detected, arguably because the BHPS disability questions lack completeness and have poor sensitivity to the cognitive dimension of disability. For the FRS and ELSA, two‐factor models can be estimated (see Tables A6, A7 and O4 of the on‐line appendix). The second factor appears to distinguish the cognitive aspect of disability for the FRS where difficulties in communication, in memory, concentration, learning or understanding and in recognizing physical danger are fairly obviously related to cognitive functioning. Since incontinence could stem from physical and/or cognitive problems, we allow for a cross‐loading between the two factors for difficulties with continence. In the ELSA, the second factor is determined from four cognitively demanding IADLs (using a map, telephone use, self‐medication and handling finances) and, as for the FRS, we allow a cross‐loading for continence. It is well known that there are limitations in the extent to which IADLs capture difficulties in cognitive functioning (Cromwell *et al*., 2003). We find that the two factors are strongly correlated (a similar result for the USA was reported by Wallace and Herzog (1995)). In the two‐factor latent disability equations (on‐line appendix Table A6) the estimated coefficients for the first factor are close to those found in the one‐factor model for the ELSA but are generally lower for the FRS, particularly for age and homeownership. Using unmatched samples, we can reject the hypothesis of equal coefficients in the FRS and ELSA models for latent disability factor 1 but not factor 2 (on‐line appendix Table A6). Results in on‐line appendix Table A7 suggest a larger role for physical than cognitive influences on AA receipt with statistically insignificant differences between the estimated coefficients in the two surveys (*p*‐values 0.140 and 0.192 respectively). The two‐factor specification confirms our previous findings on the relationship of AA receipt to socio‐economic characteristics, since tests of coefficient equality do not reject the null hypothesis that coefficients ***β*** of the observed covariates in the two‐factor models are equal to those obtained with the one‐factor specification in both surveys. The estimated coefficients of the two‐factor models are similar in size for the FRS and ELSA. On the basis of a Wald test, we reject the hypothesis of equality for the full AA coefficient vector (***β***,***γ***) (*p*‐value 0.013) but we do not reject for ***β*** alone (Wald *p*‐value 0.244). Cross‐survey differences in the magnitude of the coefficients are not large and, for practical research purposes, one would draw essentially the same conclusions from the FRS and ELSA results.

### 6.2 Alternative normalizations

The one‐factor models set out above were estimated under the normalization to 1 of the factor loading that is associated with difficulties in mobility in each survey. Here we discuss the robustness of those findings to two alternative normalizations of *η*: in the first, we constrain an alternative factor loading; in the second, we set the residual variance of *η* equal to 1.

The comparability of estimates of the disability and AA equations can be improved by normalizing the loadings of more similar questionnaire items. For instance, the FRS and ELSA have questions on the capacity to lift weights (variable LIFTING) which are arguably more similar than those on general mobility. When the factor loading for LIFTING is normalized to 1, the concordance between the FRS and ELSA disability equation and AA coefficients indeed improves, with the Wald $\chi^{2}$ *p*‐values rising to 0.271 and 0.287 respectively (one‐factor specification; unmatched samples). Details of the estimates are in the on‐line appendix, Tables O5–O7. However, the scope of this exercise is limited by the lack of a directly comparable indicator in the BHPS.

### 6.3 Proxy cases in the Family Resources Survey

Since we are forced to exclude proxy cases from the analysis of the ELSA and BHPS, we investigate the consequences of also excluding them from the FRS and dropping the proxy indicator from the disability measurement equations (see Tables O8–O10 of the on‐line appendix). This has the effect of changing slightly the factor loadings on the other indicators. Nevertheless, all factor loadings remain positive and highly significant. The largest changes in loadings are for men, where the factor loading on lifting increases from 1.005 to 1.039, whereas those for memory problems and recognizing when in danger fall from 0.420 to 0.356 and from 0.510 to 0.355 respectively. The estimated latent disability and AA receipt equations are not changed substantially. However, there are some small effects on the statistical significance of differences between the surveys in the estimated coefficients. In both the disability and the receipt‐of‐AA equations, after dropping proxy cases, the differences between the FRS and ELSA become smaller but increase slightly when the FRS is contrasted with the BHPS.

## 7 Conclusions

Our aim in this study is to contribute to the current policy debate over reform prospects for the social care system by investigating the robustness of survey‐based evidence on the targeting of public support for older people with disabilities. We have examined the three UK surveys (the FRS, ELSA and BHPS) which have been the basis for much of the empirical analysis underpinning the debate on policy on disability in the pensioner population. Despite differences between the three surveys in terms of their questionnaire content, we have found that they have a coherent story to tell about the targeting of one form of public support in relation to disability, income and other personal and household characteristics.

We also claim to offer some advance in terms of the statistical modelling methodology that is typically used in the disability research literature. Adopting a latent variable approach, we can exploit the existence of multiple—but largely arbitrary and individually unreliable—survey indicators, while avoiding the common practice of using *ad hoc* count indices as disability measures. Results confirm that the probability of receiving AA increases strongly with the severity of disability and decreases with income—especially for those in the top half of the income distribution—after allowing for the socio‐economic gradient in health that associates higher living standards with lower disability. This is important in the context of renewed suggestions that consideration be given to means testing AA (Commission on the Future of Health and Social Care in England, 2014). Contrary to some suggestions, we can say that there is no evidence of people receiving AA without any disability revealed by their survey interview. In allowing for two latent disability factors we find evidence from the FRS and ELSA that physical disability has a larger influence on AA receipt than cognitive disability. Limitations in the BHPS survey instrument meant that we could not confirm this in the BHPS. This suggests that survey designers should be concerned more to ensure that disability indicators capture a range of types of disability rather than with the merits of each individual indicator. Our use of Mahalanobis matching to improve comparability by removing differences in sample composition also provides a valuable reminder of the need to consider sample coverage as a factor when reviewing a range of research findings.

## Supporting information

‘Appendix: Additional Tables’.
