# Supplementary material for: Do household surveys give a coherent view of disability benefit targeting?: a multisurvey latent variable analysis for the older population in Great Britain
Source: J R Stat Soc Ser A Stat Soc. 2015 Mar 3;178(4):815–36. doi: 10.1111/rssa.12107 (PMC4964919; doi:10.1111/rssa.12107)
Supplement: Supplementary file 1 — ‘Appendix: Additional Tables’. [file RSSA-178-815-s001.pdf]

Appendix: Additional Tables

Table A1: Estimated 1-factor models

Disability Indicator§§	Factor loading	Standard error	Disability Indicator§§	Factor loading	Standard error
			MEN		
	FRS			*ELSA*	
MOBILITY	1	-	WALK100	1	-
LIFTING	1.005†	(0.088)	SITTING	0.386†	(0.031)
DEXTERITY	0.723†	(0.065)	CHAIR	0.581†	(0.040)
CONTINENCE	0.395†	(0.037)	CLIMBSEV	0.724†	(0.049)
COMMUNIC	0.385†	(0.042)	CLIMB1	0.990†	(0.066)
MEMORY	0.420†	(0.042)	STOOP	0.641†	(0.043)
DANGER	0.510†	(0.093)	ARMS	0.503†	(0.042)
OTHER	0.098†	(0.027)	PULL/PUSH	1.008†	(0.078)
PROXY	0.116†	(0.029)	LIFTING	0.934†	(0.066)
	BHPS		COIN	0.379†	(0.047)
HOUSEWORK	0.851†	(0.126)	DRESSING	0.661†	(0.048)
STAIRS	0.959†	(0.129)	WALKING	1.052†	(0.134)
DRESS	0.660†	(0.114)	BATH	0.863†	(0.068)
WALKING	1	-	EATING	0.596†	(0.087)
STAIRS	1.112†	(0.180)	BED	0.879†	(0.085)
MOBILITY	1.358†	(0.275)	TOILET	0.738†	(0.091)
BED	1.346†	(0.259)	CONTINENCE	0.299†	(0.030)
NAILS	0.585†	(0.085)	MAP	0.406†	(0.049)
BATH	1.001†	(0.171)	MEAL	0.806†	(0.101)
ROAD	1.151†	(0.176)	SHOPPING	1.018†	(0.084)
			PHONE	0.358†	(0.046)
			MEDICATION	0.477†	(0.071)
			HOUSEWORK	1.132†	(0.086)
			MONEY	0.453†	(0.057)
			WOMEN		
	FRS			*ELSA*	
MOBILITY	1	-	WALK100	1	-
LIFTING	1.186†	(0.102)	SITTING	0.399†	(0.029)
DEXTERITY	0.643†	(0.047)	CHAIR	0.532†	(0.033)
CONTINENCE	0.431†	(0.035)	CLIMBSEV	0.671†	(0.043)
COMMUNIC	0.365†	(0.037)	CLIMB1	0.899†	(0.053)
MEMORY	0.416†	(0.036)	STOOP	0.653†	(0.040)
DANGER	0.426†	(0.052)	ARMS	0.500†	(0.035)
OTHER	0.060‡	(0.024)	PULL/PUSH	0.899†	(0.056)
PROXY	0.121†	(0.024)	LIFTING	0.900†	(0.058)
	BHPS		COIN	0.433†	(0.037)
HOUSEWORK	0.968†	(0.149)	DRESSING	0.650†	(0.042)
STAIRS	1.201†	(0.168)	WALKING	0.959†	(0.090)
DRESS	0.910†	(0.167)	BATH	0.722†	(0.047)
WALKING	1	-	EATING	0.428†	(0.055)
STAIRS	0.911†	(0.129)	BED	0.686†	(0.054)
MOBILITY	1.066†	(0.164)	TOILET	0.577†	(0.051)
BED	0.965†	(0.151)	CONTINENCE	0.251†	(0.022)
NAILS	0.582†	(0.080)	MAP	0.343†	(0.029)
BATH	0.777†	(0.112)	MEAL	0.811†	(0.074)
ROAD	1.110†	(0.163)	SHOPPING	1.135†	(0.080)
			PHONE	0.327†	(0.045)
			MEDICATION	0.479†	(0.073)
			HOUSEWORK	0.926†	(0.061)
			MONEY	0.479†	(0.048)

Statistical significance: † $p < 0.01$; ‡ $p < 0.05$; § $p < 0.1$. §§ A more detailed description for each D_j^s indicator can be found in Online Appendix Table O1.

Table A2: Estimates of the disability equation in weighted samples

	Coefficients			Tests and coefficient differences		
Covariates	FRS	ELSA	BHPS	FRS-ELSA	FRS-BHPS	ELSA-BHPS
Spline age 65-73	0.041†	0.033†	0.136†	0.007	-0.096†	-0.103†
	(0.013)	(0.013)	(0.038)	(0.018)	(0.041)	(0.04)
Spline from age 73+	0.093†	0.101†	0.119†	-0.008	-0.027	-0.019
	(0.008)	(0.008)	(0.02)	(0.011)	(0.022)	(0.021)
Post-compulsory education	-0.275†	-0.306†	-0.214	0.031	-0.061	-0.092
	(0.067)	(0.063)	(0.155)	(0.092)	(0.169)	(0.167)
Income spline to median	-0.138†	-0.055	-0.162§	-0.083	0.024	0.108
	(0.045)	(0.054)	(0.096)	(0.07)	(0.106)	(0.11)
Income spline from median	-0.354†	-0.276†	-0.599†	-0.078	0.246	0.323
	(0.086)	(0.075)	(0.218)	(0.114)	(0.235)	(0.231)
Outright owner	-0.369†	-0.482†	-0.14	0.113	-0.229	-0.342‡
	(0.065)	(0.066)	(0.16)	(0.093)	(0.173)	(0.173)
Variance (σ_v^2)	3.004†	2.608†	3.376†	0.396	-0.372	-0.768
	(0.281)	(0.238)	(0.823)	(1.075)	(0.428)	(0.896)
	Sample size			*Coefficient equality $\chi^2(6)$*		
	6,744	5,142	1,042	3.701	10.579	14.318‡

Statistical significance of the coefficient, t-test cross-sample coefficient difference and χ^2 statistic:
† $p < 0.01$; ‡ $p < 0.05$; § $p < 0.1$. Standard Errors in parenthesis.

Table A3: Estimates of the AA receipt equation in weighted samples

	Coefficients			Coefficient differences		
Covariates	FRS	ELSA	BHPS	FRS-ELSA	FRS-BHPS	ELSA-BHPS
Latent disability η	0.569†	0.467†	0.505†	0.101§	0.064	-0.037
	(0.042)	(0.035)	(0.092)	(0.055)	(0.101)	(0.099)
Female	0.144‡	0.238†	-0.047	-0.095	0.19	0.285
	(0.066)	(0.074)	(0.184)	(0.099)	(0.195)	(0.198)
Spline age 65-73	-0.043†	-0.036†	-0.088†	-0.007	0.045§	0.052‡
	(0.008)	(0.007)	(0.022)	(0.011)	(0.023)	(0.023)
Spline from age 73+	0.056†	0.041†	0.022	0.015	0.034‡	0.019
	(0.006)	(0.007)	(0.016)	(0.009)	(0.017)	(0.017)
Post- compulsory education	-0.148‡	-0.232†	-0.099	0.084	-0.049	-0.133
	(0.066)	(0.072)	(0.155)	(0.098)	(0.168)	(0.171)
(ln) income spline to median	-0.013	-0.071	0.002	0.057	-0.015	-0.072
	(0.049)	(0.053)	(0.097)	(0.072)	(0.109)	(0.111)
(ln) income spline from median	-0.432†	-0.405†	-0.375	-0.027	-0.056	-0.029
	(0.12)	(0.152)	(0.25)	(0.193)	(0.277)	(0.292)
Outright owner	-0.135‡	-0.019	-0.244	-0.116	0.109	0.225
	(0.063)	(0.074)	(0.171)	(0.097)	(0.183)	(0.187)
Married/cohabiting	-0.038	0.087	-0.105	-0.126	0.067	0.192
	(0.066)	(0.077)	(0.196)	(0.102)	(0.207)	(0.21)
$\chi^2(9)$ test of coefficient equality				13.015	17.027‡	13.457

Statistical significance of the coefficient, t-test cross-sample coefficient difference and χ^2 statistic:
† $p < 0.01$; ‡ $p < 0.05$; § $p < 0.1$. Standard Errors in parenthesis.

Table A4: Estimates of the disability equation in matched samples

Covariate	Coefficient Estimates (standard errors)			
FRS sample composition	***ELSA matched to FRS***		***BHPS matched to FRS***	
	FRS	ELSA	FRS	BHPS
Spline age 65-73	0.047<br>(0.016)	0.036<br>(0.013)	0.073<br>(0.085)	0.142<br>(0.038)
Spline age 73+	0.090<br>(0.010)	0.098<br>(0.008)	0.077<br>(0.034)	0.119<br>(0.020)
Post- compulsory education	-0.182<br>(0.082)	-0.231<br>(0.066)	-0.001<br>(0.264)	-0.090<br>(0.161)
Income spline to median	-0.258<br>(0.097)	-0.113<br>(0.094)	-0.662<br>(0.667)	-0.925<br>(0.381)
Income spline from median	-0.314<br>(0.122)	-0.391<br>(0.089)	-0.308<br>(0.323)	-0.469<br>(0.251)
outright owner	-0.447<br>(0.082)	-0.491<br>(0.068)	-0.146<br>(0.167)	-0.226<br>(0.167)
χ^2 (6) *for coefficient equality*	1.924		5.548	
Sample size	4,587		973	
ELSA sample composition	***FRS matched to ELSA***		***BHPS matched to ELSA***	
	FRS	ELSA	ELSA	BHPS
Spline age 65-73	0.033<br>(0.016)	0.037<br>(0.013)	0.061<br>(0.031)	0.072<br>(0.035)
Spline age 73+	0.096<br>(0.010)	0.098<br>(0.008)	0.082‡<br>(0.016)	0.128‡<br>(0.022)
Post- compulsory education	-0.205<br>(0.079)	-0.271<br>(0.067)	-0.043<br>(0.143)	-0.257<br>(0.171)
Income spline to median	-0.125<br>(0.084)	-0.093<br>(0.096)	-0.284<br>(0.190)	-0.608<br>(0.382)
Income spline from median	-0.340<br>(0.118)	-0.362<br>(0.090)	-0.245<br>(0.195)	-0.512<br>(0.268)
outright owner	-0.437<br>(0.079)	-0.524<br>(0.069)	-0.442<br>(0.148)	-0.230<br>(0.164)
χ^2 (6) *for coefficient equality*	1.548		6.241	
Sample size	4,596		850	
BHPS sample composition	***FRS matched to BHPS***		***ELSA matched to BHPS***	
	FRS	BHPS	ELSA	BHPS
Spline age 65-73	0.040‡<br>(0.039)	0.143‡<br>(0.037)	0.044‡<br>(0.034)	0.133‡<br>(0.041)
Spline age 73+	0.089<br>(0.021)	0.116<br>(0.020)	0.089<br>(0.019)	0.112<br>(0.021)
Post- compulsory education	-0.075<br>(0.167)	-0.053<br>(0.156)	0.112<br>(0.159)	-0.091<br>(0.174)
Income spline to median	-0.444<br>(0.425)	-0.941<br>(0.367)	0.138<br>(0.296)	-0.296<br>(0.266)
Income spline from median	-0.403<br>(0.252)	-0.423<br>(0.249)	-0.606<br>(0.275)	-0.551<br>(0.301)
outright owner	-0.457<br>(0.182)	-0.209<br>(0.161)	-0.648<br>(0.172)	-0.318<br>(0.183)
χ^2 (6) *for coefficient equality*	7.681		9.870	
Sample size	966		791	

Note: Significance of t-test cross-sample coefficient difference and χ^2 statistic: † p < 0.01; ‡ p < 0.05; § p < 0.1.

Table A5: Estimates of the AA receipt equation in matched samples

Covariate	Coefficient Estimates (standard errors)			
FRS sample composition	***ELSA matched to FRS***		***BHPS matched to FRS***	
	FRS	ELSA	FRS	BHPS
Latent disability η	0.550	0.498	0.622	0.517
	(0.047)	(0.038)	(0.117)	(0.094)
Female	0.031	0.179	-0.037	-0.128
	(0.083)	(0.080)	(0.181)	(0.186)
Spline age 65-73	-0.031	-0.025	-0.004	0.001
	(0.010)	(0.009)	(0.058)	(0.036)
Spline age 73+	0.062	0.050	0.023	0.025
	(0.008)	(0.007)	(0.021)	(0.016)
Post- compulsory education	-0.119	-0.209	-0.107	0.146
	(0.080)	(0.080)	(0.179)	(0.169)
Income spline to median	-0.125	-0.203	-0.349	-0.688
	(0.081)	(0.086)	(0.434)	(0.360)
Income spline from median	-0.398	-0.492	-0.644	-0.304
	(0.169)	(0.200)	(0.339)	(0.267)
outright owner	-0.113	0.010	-0.223	-0.297
	(0.077)	(0.078)	(0.173)	(0.171)
Married/Cohabiting	-0.010	0.079	0.110	-0.047
	(0.082)	(0.084)	(0.179)	(0.196)
χ^2 (9) *for coefficient equality*	6.447		3.000	
Sample size	4,587		973	
ELSA sample composition	***FRS matched to ELSA***		***BHPS matched to ELSA***	
	FRS	ELSA	ELSA	BHPS
Latent disability η	0.581	0.480	0.658	0.508
	(0.051)	(0.038)	(0.119)	(0.101)
Female	0.084	0.172	0.420	0.025
	(0.082)	(0.080)	(0.219)	(0.198)
Spline age 65-73	-0.028	-0.027	-0.037	-0.003
	(0.010)	(0.009)	(0.026)	(0.032)
Spline age 73+	0.057	0.050	0.057‡	0.021‡
	(0.008)	(0.007)	(0.019)	(0.017)
Post- compulsory education	-0.139	-0.207	-0.542	0.075
	(0.082)	(0.080)	(0.209)	(0.180)
Income spline to median	-0.154	-0.184	-0.241	-0.388
	(0.080)	(0.084)	(0.207)	(0.196)
Income spline from median	-0.415	-0.530	-0.525	-0.232
	(0.170)	(0.201)	(0.449)	(0.311)
outright owner	-0.089	0.027	-0.017	-0.251
	(0.078)	(0.078)	(0.192)	(0.178)
Married/Cohabiting	-0.066	0.084	0.023	-0.275
	(0.082)	(0.082)	(0.224)	(0.199)
χ^2 (9) *for coefficient equality*	7.590		11.522	
Sample size	4,596		850	
BHPS sample composition	***FRS matched to BHPS***		***ELSA matched to BHPS***	
	FRS	BHPS	ELSA	BHPS
Latent disability η	0.519	0.530	0.566	0.510
	(0.098)	(0.096)	(0.100)	(0.103)
Female	-0.115	-0.131	0.059	-0.128
	(0.171)	(0.184)	(0.202)	(0.184)
Spline age 65-73	-0.005	0.001	-0.038	-0.047
	(0.032)	(0.035)	(0.023)	(0.030)
Spline age 73+	0.048	0.026	0.057	0.032
	(0.017)	(0.016)	(0.017)	(0.017)
Post- compulsory education	-0.076	0.147	-0.388	0.050
	(0.171)	(0.171)	(0.210)	(0.175)
Income spline to median	-0.223	-0.692	-0.265	-0.381
	(0.335)	(0.360)	(0.207)	(0.206)
Income spline from median	-0.524	-0.334	0.131	-0.318
	(0.374)	(0.27)	(0.383)	(0.308)
outright owner	-0.259	-0.302	0.011	-0.289
	(0.176)	(0.171)	(0.202)	(0.183)
Married/Cohabiting	-0.021	-0.031	-0.095	-0.103
	(0.200)	(0.195)	(0.207)	(0.198)
χ^2 (9) *for coefficient equality*	3.445		6.619	
Sample size	966		791	

Note: Significance of t-test cross-sample coefficient difference and χ^2 statistic: † $p < 0.01$; ‡ $p < 0.05$; § $p < 0.1$.

Table A6: Estimates of the latent disability equation for the FRS and ELSA 2-factor models

Covariates	η1			η2		
	FRS	ELSA	Tests and coefficient differences	FRS	ELSA	Tests and coefficient differences
Spline age 65-73	0.033†	0.035†	-0.002	0.025	-0.015	0.040§
	(0.003)	(0.011)	(0.011)	(0.016)	(0.013)	(0.021)
Spline from age 73+	0.064†	0.095†	-0.031†	0.079†	0.071†	0.008
	(0.005)	(0.007)	(0.009)	(0.008)	(0.009)	(0.012)
Post-compulsory education	-0.237†	-0.276†	0.039	-0.142§	-0.241†	0.100
	(0.051)	(0.058)	(0.077)	(0.075)	(0.069)	(0.102)
Income spline to median	-0.103†	-0.039	-0.063	-0.175†	-0.119‡	-0.056
	(0.037)	(0.051)	(0.063)	(0.038)	(0.047)	(0.061)
Income spline from median	-0.293†	-0.305†	0.013	-0.086	-0.170§	0.084
	(0.071)	(0.070)	(0.100)	(0.102)	(0.090)	(0.136)
Outright owner	-0.334†	-0.484†	0.150§	-0.120§	-0.135‡	0.015
	(0.053)	(0.062)	(0.081)	(0.072)	(0.061)	(0.095)
χ^2(6) coefficient equality			19.616†			7.423
Sample size	6744			5142		

Statistical significance of the coefficient, t-test cross-sample coefficient difference and χ^2 statistic:
† p < 0.01; ‡ p < 0.05; § p < 0.1. Standard Errors in parenthesis.

Table A7: Estimates of the AA receipt equation for the FRS and ELSA 2-factor models

Covariates	FRS		ELSA		tests and coefficient differences	
Latent disability η_1	0.508†	(0.039)	0.419†	(0.045)	0.089	(0.060)
Latent disability η_2	0.295†	(0.046)	0.164§	(0.089)	0.131	(0.100)
Female	-0.043†	(0.006)	-0.032†	(0.007)	-0.012	(0.010)
Spline age 65-73	0.055†	(0.006)	0.042†	(0.007)	0.013	(0.009)
Spline from age 73+	-0.166‡	(0.065)	-0.222†	(0.072)	0.056	(0.097)
Post- compulsory education	-0.001	(0.048)	-0.078	(0.050)	0.077	(0.069)
(ln) income spline to median e	-0.406†	(0.120)	-0.421†	(0.153)	0.015	(0.195)
(ln) income spline from median	-0.149‡	(0.063)	-0.015	(0.072)	-0.135	(0.096)
Outright owner	-0.079	(0.065)	0.084	(0.077)	-0.163	(0.101)
Married/cohabiting	0.183‡	(0.072)	0.271†	(0.075)	-0.088	(0.104)
χ^2(10) coefficient equality					22.477‡	
Sample size	6744		5142			

Statistical significance of the coefficient, t-test cross-sample coefficient difference and χ^2 statistic:
† p < 0.01; ‡ p < 0.05; § p < 0.1. Standard Errors in parenthesis.

Online Appendix: Further Tables and Identification proof

Table O1: Survey specific functional limitations indicators D

Data Source:		Not receiving AA		Receiving AA		Non-recipient/ recipient difference (unweighted)[†]
		un-weighted mean	weighted mean	un-weighted mean	weighted mean	
FRS: *Has difficulty with:*						
MOBILITY	mobility (moving about)	0.251	0.254	0.814	0.813	-0.563
LIFTING	lifting, carrying or moving objects	0.221	0.221	0.745	0.749	-0.524
DEXTERITY	manual dexterity using hands for everyday tasks	0.077	0.077	0.396	0.400	-0.319
CONTINENCE	with continence (bladder control)	0.055	0.056	0.237	0.235	-0.182
COMMUNICATION	communication (speech, hearing or eyesight)	0.039	0.040	0.204	0.200	-0.165
MEMORY	memory/concentration/learning/understanding	0.049	0.050	0.252	0.255	-0.203
KNOWING DANGER	recognising when in physical danger	0.005	0.005	0.068	0.069	-0.062
OTHER	other area of life	0.040	0.040	0.092	0.091	-0.053
PROXY	interviewed by proxy	0.059	0.059	0.121	0.131	-0.063
Observations		*6,093*		*651*		
ELSA: *Has difficulty with:*						
WALKING 100 YDS	walking 100 yards	0.117	0.121	0.572	0.582	-0.455
SITTING 2 HRS	sitting for about two hours	0.126	0.126	0.285	0.279	-0.158
CHAIR TRANSFERS	getting up from a chair after sitting for long periods	0.282	0.285	0.626	0.618	-0.344
STAIRS (several flights)	climbing several flights of stairs without resting	0.424	0.429	0.821	0.822	-0.397
STAIRS (1 flights)	climbing one flight of stairs without resting	0.161	0.167	0.650	0.653	-0.489
STOOPING	stooping, kneeling, or crouching	0.411	0.415	0.791	0.798	-0.381
REACHING	reaching or extending arms above shoulder level	0.103	0.105	0.344	0.339	-0.241
PULL/PUSHING	pulling or pushing large objects e.g. living room chair	0.183	0.189	0.675	0.686	-0.492
LIFTING	lifting/carrying weights over 10 lbs, e.g. heavy bag	0.281	0.288	0.797	0.806	-0.516
PICKING-UP COIN	picking up a 5p coin from a table	0.049	0.050	0.241	0.249	-0.192
DRESSING	ADL:dressing, including putting on shoes an	0.126	0.128	0.472	0.460	-0.346
WALKING	ADL:walking across a room	0.025	0.027	0.203	0.211	-0.178
BATHING	ADL:bathing or showering	0.128	0.132	0.566	0.568	-0.438
FEEDING	ADL:eating, such as cutting up your food	0.012	0.012	0.092	0.095	-0.08
BED TRANSFERS	ADL:getting in or out of bed	0.044	0.045	0.287	0.280	-0.243
USING TOILET	ADL:using the toilet, including getting up	0.029	0.030	0.179	0.179	-0.15
CONTINENCE	Problem with continence	0.157	0.158	0.336	0.338	-0.179
USING MAP	IADL:using a map to figure out how to get around	0.057	0.061	0.222	0.240	-0.165
PREP HOT MEAL	IADL:preparing a hot meal	0.029	0.031	0.282	0.291	-0.253
SHOPPING	IADL:shopping for groceries	0.083	0.088	0.504	0.515	-0.422
PHONING	IADL:making telephone calls	0.020	0.022	0.095	0.095	-0.075
MEDICATION	IADL:taking medications	0.010	0.011	0.084	0.086	-0.073
HOUSEWORK	IADL:doing work around the house or garden	0.159	0.163	0.650	0.660	-0.491
MANAGING MONEY	IADL: managing money, e.g. paying bills	0.023	0.025	0.154	0.162	-0.131
Observations		*4,773*		*369*		
BHPS: *Health hinders:*						
HOUSEWORK	doing the housework	0.089	0.095	0.573	0.557	-0.484
CLIMBING STAIRS	climbing the stairs	0.105	0.114	0.600	0.601	-0.495
DRESSING	getting dressed	0.036	0.038	0.173	0.185	-0.137
WALKING > 10 mins	walking more than 10 mins	0.094	0.097	0.520	0.526	-0.426
How manages...(6-point scale)						
STAIRS	Stairs	1.856	1.914	3.920	3.830	-2.064
AROUND HOUSE	getting around house	1.350	1.367	2.613	2.551	-1.264
BED TRANSFERS	getting in/out bed	1.360	1.378	2.547	2.525	-1.187
CUTTING TOENAILS	cutting toenails	2.555	2.643	4.920	4.915	-2.365
BATHING	bathing/showering	1.572	1.626	3.280	3.286	-1.708
WALKING DOWN ROAD	walking down road	1.678	1.720	3.773	3.739	-2.095
Observations		*967*		*75*		

[†] All differences are significantly different from 0 at the 1% level.

Table O2: Sample means of SES and AA receipt in FRS, ELSA and BHPS

	FRS		ELSA		BHPS	
	mean	sd	mean	sd	mean	sd
			Unweighted			
Female	0.559	*0.497*	0.557	*0.497*	0.560	*0.497*
Age†	74		73		74	
Post-compulsory education	0.505	*0.500*	0.539	*0.499*	0.513	*0.500*
Ln pre-benefit equivalised income‡	6.454	*0.806*	6.412	*0.751*	6.551	*0.732*
Outright owner	0.664	*0.472*	0.690	*0.463*	0.701	*0.458*
Married/cohabiting	0.579	*0.494*	0.565	*0.496*	0.553	*0.497*
Receives AA	0.097	*0.295*	0.072	*0.258*	0.072	*0.259*
			Weighted			
Female	0.555	0.497	0.571	0.495	0.561	0.497
Age†	74		74		74	
Post-compulsory education	0.513	0.500	0.522	0.500	0.495	0.500
Ln pre-benefit equivalised income‡	6.463	0.826	6.391	0.754	6.521	0.746
Outright owner	0.677	0.468	0.682	0.466	0.672	0.470
Married/cohabiting	0.573	0.495	0.548	0.498	0.538	0.499
Receives AA	0.094	0.292	0.077	0.266	0.079	0.270
Observations	6,746		5,142		1,042	

Notes: †To protect confidentiality, FRS and ELSA release data with a top-coding at the age of 80 and 90, respectively. Therefore, we report median rather than mean values. ‡ Household income excludes disability and means tested benefits and it has been equivalised using the modified OECD equivalence scale.

Table O3: Sample means of SES and AA receipt in matched samples

	FRS		ELSA		FRS		BHPS	
	mean	sd	mean	sd	mean	sd	mean	sd
FRS sample composition:	*ELSA matched to FRS*				*BHPS matched to FRS*			
	FRS		ELSA		FRS		BHPS	
Female	0.561	*0.496*	0.561	*0.496*	0.566	*0.496*	0.566	*0.496*
Age†	73		73		74		74	
Post-compulsory schooling	0.530	*0.499*	0.530	*0.499*	0.506	*0.500*	0.506	*0.500*
ln pre-benefit equivalised income‡	6.457	*0.582*	6.456	*0.582*	6.576	*0.503*	6.600	*0.500*
Accommodation own it outright	0.690	*0.462*	0.690	*0.462*	0.716	*0.451*	0.716	*0.451*
Married/cohabiting	0.572	*0.495*	0.572	*0.495*	0.565	*0.496*	0.565	*0.496*
Receives AA	0.088	*0.283*	0.071	*0.257*	0.094	*0.291*	0.072	*0.259*
Observations	4,587				973			
ELSA sample composition:	*FRS matched to ELSA*				*BHPS matched to ELSA*			
	FRS		ELSA		ELSA		BHPS	
Female	0.562	*0.496*	0.562	*0.496*	0.575	*0.495*	0.575	*0.495*
Age†	73		73		74		74	
Post-compulsory schooling	0.531	*0.499*	0.531	*0.499*	0.504	*0.500*	0.504	*0.500*
ln pre-benefit equivalised income‡	6.458	*0.578*	6.455	*0.582*	6.563	*0.513*	6.533	*0.527*
accommodation own it outright	0.690	*0.463*	0.690	*0.463*	0.720	*0.449*	0.720	*0.449*
Married/cohabiting	0.574	*0.495*	0.574	*0.495*	0.552	*0.498*	0.552	*0.498*
Receives AA	0.089	*0.284*	0.070	*0.255*	0.072	*0.258*	0.066	*0.248*
Observations	4,596				850			
BHPS sample composition:	*FRS matched to BHPS*				*ELSA matched to BHPS*			
	FRS		BHPS		ELSA		BHPS	
Female	0.565	*0.496*	0.565	*0.496*	0.564	*0.496*	0.564	*0.496*
Age†	74		74		74		74	
Post-compulsory schooling	0.505	*0.500*	0.505	*0.500*	0.497	*0.500*	0.497	*0.500*
ln pre-benefit equivalised income‡	6.575	*0.499*	6.599	*0.496*	6.488	*0.496*	6.513	*0.500*
accommodation own it outright	0.716	*0.451*	0.716	*0.451*	0.718	*0.450*	0.718	*0.450*
Married/cohabiting	0.566	*0.496*	0.566	*0.496*	0.550	*0.498*	0.550	*0.498*
Receives AA	0.085	*0.279*	0.072	*0.259*	0.068	*0.252*	0.078	*0.269*
Observations	966				791			

Notes: Based on unweighted selected samples. † To protect confidentiality, FRS and ELSA release data with a top-coding at the age of 80 and 90, respectively. Therefore, we report median rather than mean values. ‡ Household income excludes disability and means tested benefits and it has been equivalised using the modified OECD equivalence scale.

Table O4: Factor loadings for the FRS and ELSA 2-factor models and squared correlations of disability indicators with latent indexes (η_q)

Functional limitation indicator	Male		Female	
	Factor 1 (η_1)	Factor 2 (η_2)	Factor 1 (η_1)	Factor 2 (η_2)
FRS cov(η_1,η_2)	1.172		0.854	
MOBILITY	1		1	
LIFTING	1.586†		2.226†	
DEXTERITY	0.768†		0.736†	
CONTINENCE	0.315†	0.235†	0.363†	0.275†
COMMUNIC		1		1
MEMORY		0.837†		0.987†
DANGER		1.005†		1.078†
OTHER	0.009	0.144‡	-0.064	0.208†
PROXY		0.204†		0.270†
ELSA cov(η_1,η_2)	1.058		0.890	
WALK100	1		1	
SITTING	0.394†		0.409†	
CHAIR	0.593†		0.545†	
CLIMBSEV	0.736†		0.689†	
CLIMB1	1.014†		0.918†	
STOOP	0.657†		0.669†	
ARMS	0.511†		0.511†	
PULL/PUSH	1.025†		0.921†	
LIFTING	0.954†		0.919†	
COIN	0.383†		0.44†	
DRESSING	0.673†		0.665†	
WALKING	1.082†		0.980†	
BATH	0.879†		0.736†	
EATING	0.586†		0.431†	
BED	0.897†		0.705†	
TOILET	0.751†		0.592†	
CONTINENCE	0.196†	0.235‡	0.275†	-0.047
MAP		1.052†		1.031†
MEAL				
SHOPPING	0.999†		1.129†	
PHONE		1		1
MEDICATION		1.231†		1.319†
HOUSEWORK	1.137†		0.938†	
MONEY		1.25†		1.731†

Statistical significance of the factor loadings: † $p < 0.01$; ‡ $p < 0.05$; § $p < 0.1$.

Table O5: Factor loadings for the FRS and ELSA 1-factor models with alternative factor loading constraints

FRS					ELSA				
Disability Indicator	Factor loading (St. err.)				Disability Indicator	Factor loading (St. err.)			
	Men		Women			Men		Women	
MOBILITY	0.849†	(0.072)	0.962†	(0.077)	WALKING 100 YDS	1.118†	(0.079)	1.077†	(0.039)
LIFTING	1	-	1	-	SITTING 2 HRS	0.422†	(0.034)	0.436†	(0.030)
DEXTERITY	0.663†	(0.058)	0.579†	(0.040)	CHAIR TRANSFERS	0.635†	(0.042)	0.582†	(0.035)
CONTINENCE	0.360†	(0.035)	0.392†	(0.033)	STAIRS (several flights)	0.792†	(0.050)	0.735†	(0.042)
COMMUNIC	0.351†	(0.039)	0.333†	(0.035)	STAIRS (1 flight)	1.084†	(0.069)	0.984†	(0.058)
MEMORY	0.382†	(0.04)	0.380†	(0.035)	STOOPING	0.701†	(0.044)	0.715†	(0.040)
DANGER	0.461†	(0.086)	0.388†	(0.050)	REACHING	0.550†	(0.044)	0.547†	(0.037)
OTHER	0.089†	(0.025)	0.055‡	(0.022)	PULL/PUSHING	1.100†	(0.071)	0.987†	(0.050)
PROXY	0.105†	(0.027)	0.110†	(0.022)	LIFTING	1	-	1	-
					PICKING-UP COIN	0.415†	(0.051)	0.474†	(0.039)
					DRESSING	0.723†	(0.051)	0.711†	(0.046)
					WALK ACROSS ROOM	1.154†	(0.151)	1.048†	(0.099)
					BATHING	0.944†	(0.073)	0.790†	(0.050)
					FEEDING	0.652†	(0.093)	0.468†	(0.060)
					BED TRANSFERS	0.962†	(0.093)	0.751†	(0.058)
					USING TOILET	0.808†	(0.097)	0.631†	(0.054)
					CONTINENCE	0.327†	(0.032)	0.275†	(0.023)
					USING A MAP	0.445†	(0.052)	0.375†	(0.031)
					PREP. HOT MEAL	0.883†	(0.109)	0.886†	(0.081)
					SHOPPING	1.115†	(0.091)	1.241†	(0.086)
					PHONING	0.392†	(0.049)	0.357†	(0.049)
					MEDICATION	0.523†	(0.077)	0.524†	(0.081)
					HOUSEWORK	1.239†	(0.092)	1.014†	(0.063)
					MANAGING MONEY	0.496†	(0.061)	0.524†	(0.052)
Sample size	6,744					5,142			

Statistical significance of the factor loadings: † $p < 0.01$; ‡ $p < 0.05$; § $p < 0.1$.

Table O6: Estimates of the latent disability equation for the FRS and ELSA 1-factor models with alternative factor loading constraints

Covariates	Coefficients and Standard Errors: FRS	ELSA	Tests and coefficient differences	
Spline age 65-73	0.042† (0.014)	0.032† (0.011)	0.010	(0.018)
Spline from age 73+	0.100† (0.009)	0.090† (0.007)	0.010	(0.011)
Post-compulsory education	-0.307† (0.074)	-0.255† (0.055)	-0.052	(0.092)
Income spline to median	-0.180† (0.052)	-0.042 (0.048)	-0.137§	(0.070)
Income spline from median	-0.369† (0.094)	-0.284† (0.066)	-0.085	(0.115)
Outright owner	-0.416† (0.071)	-0.444† (0.057)	0.028	(0.092)
	Sample size		*Coefficient equality* χ^2 (6)	
	6,744	5,142	7.573	

Statistical significance of the coefficient, t-test cross-sample coefficient difference and χ^2 statistic:
† p < 0.01; ‡ p < 0.05; § p < 0.1. Standard Errors in parenthesis.

Table O7: Estimates of the AA receipt equation for the FRS and ELSA 1-factor models with alternative factor loading constraints

Covariates	Coefficients and Standard Errors: FRS	ELSA	Tests of coefficient equality: FRS-ELSA	
Latent disability η	0.516† (0.041)	0.522† (0.038)	-0.006	(0.056)
Female	0.118§ (0.065)	0.252† (0.073)	-0.134	(0.098)
Spline age 65-73	-0.040† (0.008)	-0.036† (0.007)	-0.004	(0.011)
Spline from age 73+	0.058† (0.006)	0.046† (0.007)	0.012	(0.009)
Post- compulsory education	-0.161‡ (0.065)	-0.238† (0.071)	0.077	(0.096)
(ln) income spline to median	-0.007 (0.048)	-0.092§ (0.049)	0.085	(0.069)
(ln) income spline from median	-0.390† (0.120)	-0.422† (0.154)	0.032	(0.195)
Outright owner	-0.138‡ (0.062)	-0.006 (0.071)	-0.132	(0.095)
Married/cohabiting	-0.077 (0.064)	0.087 (0.076)	-0.164	(0.100)
	Sample size		χ^2*(9) test of coefficient equality*	
	6,744	5,142	10.841	

Statistical significance of the coefficient, t-test cross-sample coefficient difference and χ^2 statistic:
† p < 0.01; ‡ p < 0.05; § p < 0.1. Standard Errors in parenthesis.

Table O8: Factor loadings for the FRS 1-factor model excluding proxy cases from the FRS sample (and the proxy indicator from the measurement model)

	FRS			
	Factor loading (St. err.)			
Disability Indicator	Men		Women	
MOBILITY	1	-	1	-
LIFTING	1.039†	(0.103)	1.203†	(0.123)
DEXTERITY	0.683†	(0.065)	0.602†	(0.049)
CONTINENCE	0.343†	(0.036)	0.426†	(0.037)
COMMUNIC	0.338†	(0.041)	0.317†	(0.036)
MEMORY	0.356†	(0.039)	0.382†	(0.036)
DANGER	0.355†	(0.091)	0.408†	(0.063)
OTHER	0.101†	(0.029)	0.068†	(0.026)
Sample size	6,308			

Statistical significance of the factor loadings: † $p < 0.01$; ‡ $p < 0.05$; § $p < 0.1$.

Table O9: Estimates of the latent disability equations obtained by dropping proxy cases from the FRS sample (and the proxy indicator from the measurement model)

	Coefficients and Standard Errors			Tests and coefficient differences		
Covariates	FRS	ELSA§§	BHPS§§	FRS-ELSA	FRS-BHPS	ELSA-BHPS§§
Spline age 65-73	0.039†	0.035†	0.127†	0.003	-0.089‡	-0.092†
	(0.014)	(0.012)	(0.036)	(0.018)	(0.038)	(0.038)
Spline from age 73+	0.084†	0.099†	0.128†	-0.015	-0.044‡	-0.029
	(0.008)	(0.008)	(0.020)	(0.011)	(0.022)	(0.022)
Post-compulsory education	-0.301†	-0.280†	-0.182	-0.021	-0.119	-0.097
	(0.068)	(0.061)	(0.149)	(0.091)	(0.164)	(0.161)
Income spline to median	-0.114‡	-0.046	-0.172§	-0.068	0.057	0.125
	(0.052)	(0.052)	(0.104)	(0.074)	(0.116)	(0.116)
Income spline from median	-0.317†	-0.310†	-0.558†	-0.007	0.241	0.248
	(0.088)	(0.072)	(0.206)	(0.114)	(0.224)	(0.218)
Outright owner	-0.389†	-0.487†	-0.185	0.098	-0.204	-0.302§
	(0.067)	(0.064)	(0.151)	(0.092)	(0.165)	(0.163)
	Sample size			*Coefficient equality* χ^2 (6)		
	6,308	5,142	1,042	3.411	13.27‡	14.139‡

Statistical significance of the coefficient, t-test cross-sample coefficient difference and χ^2 statistic: † $p < 0.01$; ‡ $p < 0.05$; § $p < 0.1$. Standard Errors in parenthesis. §§ Estimates are the same reported in Table 2.

Table O10: Estimates of the AA receipt equations obtained by dropping PROXY cases from the FRS sample (and the proxy indicator from the measurement model)

	Coefficients and Standard Errors			Tests of coefficient equality		
Covariates	FRS	ELSA[§§]	BHPS[§§]	FRS-ELSA	FRS-BHPS	ELSA-BHPS[§§]
Latent disability η	0.573[†]	0.477[†]	0.538[†]	0.096[§]	0.036	-0.06
	(0.043)	(0.035)	(0.095)	(0.056)	(0.104)	(0.101)
Female	0.156[‡]	0.251[†]	-0.068	-0.095	0.224	0.319[§]
	(0.069)	(0.073)	(0.172)	(0.101)	(0.185)	(0.187)
Spline age 65-73	-0.041[†]	-0.036[†]	-0.084[†]	-0.004	0.043[§]	0.048[‡]
	(0.009)	(0.007)	(0.021)	(0.011)	(0.023)	(0.022)
Spline from age 73+	0.059[†]	0.046[†]	0.028[§]	0.014	0.031[§]	0.017
	(0.006)	(0.007)	(0.015)	(0.009)	(0.016)	(0.016)
Post- compulsory education	-0.153[‡]	-0.238[†]	-0.070	0.085	-0.083	-0.167
	(0.069)	(0.071)	(0.155)	(0.099)	(0.170)	(0.171)
(ln) income spline to median	-0.044	-0.092[§]	-0.041	0.048	-0.002	-0.050
	(0.063)	(0.049)	(0.090)	(0.079)	(0.109)	(0.102)
(ln) income spline from median	-0.493[†]	-0.422[†]	-0.411[§]	-0.071	-0.082	-0.011
	(0.136)	(0.154)	(0.247)	(0.205)	(0.282)	(0.291)
Outright owner	-0.137[‡]	-0.006	-0.265	-0.131	0.128	0.259
	(0.065)	(0.071)	(0.164)	(0.097)	(0.176)	(0.178)
Married/cohabiting	-0.058	0.087	-0.171	-0.145	0.112	0.257
	(0.068)	(0.076)	(0.182)	(0.102)	(0.195)	(0.198)
		Sample size			*Coefficient equality* χ^2 (9)	
	6,308	5,142	1,042	13.287	14.957[§]	14.844[§]

Statistical significance of the coefficient, t-test cross-sample coefficient difference and χ^2 statistic: † $p < 0.01$; ‡ $p < 0.05$; § $p < 0.1$. Standard Errors in parenthesis. [§§] Estimates are the same reported in Table 3.

Identification

After using equation (3) to solve out the latent disability variables η_{iq} from the model, the structure can be written in matrix form as:

$$\widetilde{\mathbf{D}} = \mathbf{\Lambda\Theta z} + \mathbf{\Lambda \upsilon} + \boldsymbol{\varepsilon} \tag{A1}$$

$$\widetilde{R} = (\boldsymbol{\beta} + \boldsymbol{\gamma\Theta})\mathbf{z} + \boldsymbol{\gamma\upsilon} + u \tag{A2}$$

where $\mathbf{\Lambda}$, $\mathbf{\Theta}$, $\boldsymbol{\beta}$ and $\boldsymbol{\gamma}$ are respectively $K_s \times Q$, $Q \times p$, $1 \times p$ and $1 \times Q$ dimensional coefficient matrices and we have omitted the individual i suffix from the covariates $\mathbf{z}$, the latent variables $\widetilde{\mathbf{D}}$, and $\widetilde{R}$ underlying the observed ordinal variables $\mathbf{D}$ and R, and the unobservable random terms $\boldsymbol{\upsilon}$, $\boldsymbol{\varepsilon}$ and u. Equations (A1)-(A2) together comprise a system of correlated reduced form (ordered) probit equations, from which we can identify the following coefficient matrices and residual covariances:

$$\mathbf{B}_1 = \mathbf{\Lambda\Theta} \tag{A3}$$

$$\mathbf{B}_2 = \boldsymbol{\beta} + \boldsymbol{\gamma\Theta} \tag{A4}$$

$$\mathbf{C}_{11} = \mathbf{\Lambda\Omega\Lambda}' + \mathbf{\Sigma} \tag{A5}$$

$$\mathbf{C}_{22} = \boldsymbol{\gamma\Omega\gamma}' + \boldsymbol{\gamma\delta} + \sigma^2 \tag{A6}$$

$$\mathbf{C}_{12} = \mathbf{\Lambda\Omega}\boldsymbol{\gamma}' + \mathbf{\Lambda}\boldsymbol{\delta} \tag{A7}$$

where $\mathbf{\Omega}$ is the covariance matrix of $\boldsymbol{\upsilon}$, $\mathbf{\Sigma}$ is the diagonal covariance matrix of $\boldsymbol{\varepsilon}$, $\boldsymbol{\delta}$ is the vector of covariances between $\boldsymbol{\upsilon}$ and u, and σ^2 is the variance of u.

Some normalisations are necessary, because the observed variables $\mathbf{D}$ and R do not reveal the scale of $\widetilde{\mathbf{D}}$ and $\widetilde{R}$ and because the latent $\boldsymbol{\eta}$ can be replaced by arbitrary linear combinations with the loadings $\mathbf{\Theta}$ and $\boldsymbol{\gamma}$ transformed accordingly. Without loss of generality, we resolve these indeterminacies by setting C_{22} and the diagonal elements of C_{11} to unity and by imposing the restrictions:

$$\mathbf{\Lambda} = \begin{pmatrix} \mathbf{I} \\ \mathbf{\Lambda}_2 \end{pmatrix} \tag{A8}$$

Given these normalisations, the first Q rows of $\mathbf{B}_1$ identify $\mathbf{\Theta}$. Provided the rank of $\mathbf{\Theta}$ is Q, $\mathbf{\Lambda}_2$ can then be found by solving the last K_s -Q equations in (A3). This rank condition implies that the Q latent factors in the measurement equations (1) cannot be replaced by a smaller number of linear combinations of the factors.

Now consider identification of $\mathbf{\Omega}$. Write the vector of Q diagonal elements of $\mathbf{\Omega}$ as $\boldsymbol{\omega}_d$ and the vector of $(Q$-1)/2 sub-diagonal elements as $\boldsymbol{\omega}_s$. We can construct an identity: vec($\mathbf{\Omega}$)

$= \mathbf{S}_d \boldsymbol{\omega}_d + \mathbf{S}_s \boldsymbol{\omega}_s$ where $\mathbf{S} = (\mathbf{S}_d\ \mathbf{S}_s)$ is a $Q^2 \times Q(Q+1)/2$ permutation matrix containing 1s and 0s and vec(.) is the operation of stacking the rows of a matrix into a column vector. Let $\mathbf{C}_{11}^{1,1}$ be the leading $Q \times Q$ block of $\mathbf{C}_{11}$ and note that Σ is diagonal so that $\mathbf{S}'_s \mathrm{vec}\left(\mathbf{C}_{11}^{1,1}\right) = \boldsymbol{\omega}_s$. This determines the off-diagonal elements of $\boldsymbol{\omega}$. Now let $\mathbf{C}_{11}^{1,2}$ be the submatrix of $\mathbf{C}_{11}$ containing elements from the first Q rows and last K_s-Q columns: then $\mathbf{C}_{11}^{1,2} = \boldsymbol{\Omega}\boldsymbol{\Lambda}_2'$ and, if c_{qj} is the typical element of $\mathbf{C}_{11}^{1,2}$, each of the ω_{qq} can be deduced as $\omega_{qq} = \left(c_{qj} - \sum_{r \neq q} \omega_{qr} \lambda_{jr}^s \right) / \lambda_{jq}^s$, provided there exists at least one non-zero element in the qth column of Λ_2, for each q = 1...Q. With $\boldsymbol{\Omega}$ determined, Σ is immediately given by (A5).

Without further restrictions, this is as far as we can go. Once Θ, Λ, $\boldsymbol{\Omega}$ and Σ are known, this still leaves $p + 2Q + 1$ parameters $\boldsymbol{\beta}$, γ, δ and σ^2 to be determined by the $p + Q + 1$ equations in (A4), (A6) and (A7). At least Q further restrictions are necessary. Natural possibilities are $\boldsymbol{\delta} = \mathrm{cov}(\upsilon, u) = \mathbf{0}$ or exclusion restrictions on the vector $\boldsymbol{\beta}$. The latter requires the existence of covariates that can be assumed a priori to influence disability status (relevance) but have no causal role in determining benefit receipt (validity).
